# MicroRNAs in Kidney Fibrosis and Diabetic Nephropathy: Roles on EMT and EndMT

**DOI:** 10.1155/2013/125469

**Published:** 2013-09-08

**Authors:** Swayam Prakash Srivastava, Daisuke Koya, Keizo Kanasaki

**Affiliations:** Department of Diabetology & Endocrinology, Kanazawa Medical University, Uchinada, Ishikawa 920-0293, Japan

## Abstract

MicroRNAs (miRNAs) are a family of small, noncoding RNAs that regulate gene expression in diverse biological and pathological processes, including cell proliferation, differentiation, apoptosis, and carcinogenesis. As a result, miRNAs emerged as major area of biomedical research with relevance to kidney fibrosis. Fibrosis is characterized by the excess deposition of extracellular matrix (ECM) components, which is the end result of an imbalance of metabolism of the ECM molecule. Recent evidence suggests that miRNAs participate in the fibrotic process in a number of organs including the heart, kidney, liver, and lung. Epithelial mesenchymal transition (EMT) and endothelial mesenchymal transition (EndMT) programs play vital roles in the development of fibrosis in the kidney. A growing number of the extracellular and intracellular molecules that control EMT and EndMT have been identified and could be exploited in developing therapeutics for fibrosis. This review highlights recent advances on the role of miRNAs in the kidney diseases; diabetic nephropathy especially focused on EMT and EndMT program responsible for the development of kidney fibrosis. These miRNAs can be utilized as a potential novel drug target for the studying of underlying mechanism and treatment of kidney fibrosis.

## 1. Introduction

MicroRNAs (miRNAs) are short noncoding RNAs that modulate fundamental cellular processes such as differentiation, proliferation, death, metabolism, and pathophysiology of many diseases by inhibiting target gene expression via inhibition of protein translation or by inducing mRNA degradation. By recent estimates, nearly 1000 human miRNAs target and downregulate at least 60% of human protein coding genes expressed in the genome [1]. The understandings of miRNAs in molecular mechanisms on various disease processes are now expanding day by day. In the current scenario, miRNAs play the role of conductors in the pathogenesis of fibrosis diseases. There are many literatures that organ-specific miRNAs alterations cause fibrotic disorders [2]. Fibrosis is the leading cause of organ dysfunction in diseases, either as outcome of an uncontrolled reaction to chronic tissue injury or as the primary disease itself in predisposed individuals [3]. Fibrosis of the kidney is caused by prolonged injury and dysregulation of normal wound healing process in association with an excess deposition of extracellular matrix. In such fibrotic process, kidney fibroblasts play important roles but the origin of fibroblasts remains elusive. In addition to the activation of residential fibroblasts, other important sources of fibroblasts have been proposed such as pericytes, fibrocytes, and fibroblasts originated from epithelial mesenchymal transition, endothelial mesenchymal transition. The two main loci for fibrosis in the kidney are the tubulointerstitial space and the glomerulus. Recent studies using transgenic mice have demonstrated that primary changes in glomeruli can lead to progressive glomerulosclerosis and renal failure [4]. For these reasons and knowing the multitude of pathways that miRNAs can affect, it is envisaged that investigating the roles of miRNAs in fibrosis could not only advance our understanding of the pathogenesis of this common condition but might also provide new targets for therapeutic intervention. In this review we focused on roles of miRNA biology in the kidney disease especially in epithelial mesenchymal transition (EMT) and endothelial mesenchymal transition (EndMT) programs. 

## 2. miRNA Gene and Transcription

miRNAs are single-stranded RNAs (ssRNAs) of ~22 nt in length that are generated from endogenous hairpin-shaped transcripts [5]. miRNAs function as guide molecules in posttranscriptional gene regulation by base-pairing with the target mRNAs, usually in the 3′ untranslated region (UTR). Binding of a miRNA to the target mRNA typically leads to translational repression and exonucleolytic mRNA decay, although highly complementary targets can be cleaved endonucleolytically. Over one-third of human genes are predicted to be directly targeted by miRNAs.

**Figure 1:**
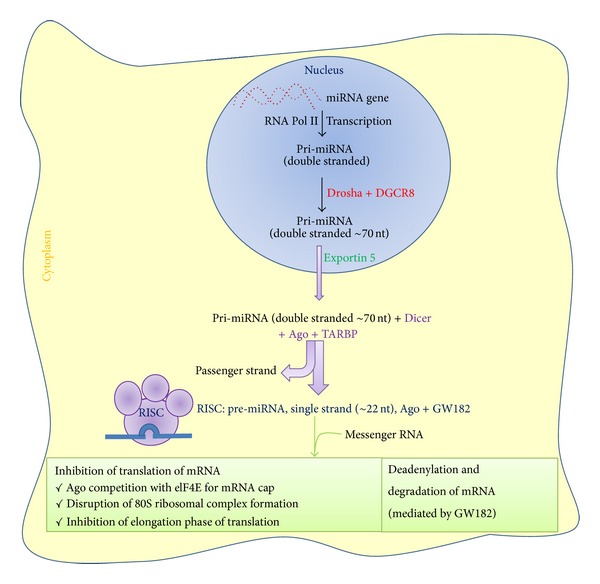
Schematic presentation of biogenesis and action of miRNAs. Ago: Argonaute; DGCR8: DiGeorge syndrome critical region 8; elF4E: eukaryotic initiation factor 4E; GW182: glycine-tryptophan protein-182; nt: nucleotides; RISC: miRNA-induced silencing complex; TARBP: transactivation-responsive RNA-binding protein.

The 1st step in miRNAs biogenesis is nuclear processing by Drosha; the primary transcripts (pri-miRNAs) that are generated by Pol II are usually several kilobases long and contain local stem-loop structures (Figure 1). The first step of miRNA maturation is cleavage at the stem of the hairpin structure, which releases a small hairpin that is termed a pre-miRNA. This reaction takes place in the nucleus by the nuclear RNase III-type protein Drosha. Drosha requires a cofactor, the DiGeorge syndrome critical region gene 8 (DGCR8) protein in humans (Pasha in *D. melanogaster* and *C. elegans*) [6]. Together with DGCR8 (or Pasha), Drosha forms a large complex known as the microprocessor complex, which is ~500 kDa in *D. melanogaster* and ~650 kDa in humans [6]. Drosha and DGCR8 are conserved only in animals. The 2nd step in biogenesis is the nuclear export by the exportin 5. The trimmed precursor (pre-miRNA) hairpins from both canonical and noncanonical miRNA pathways are then transported by an exportin 5 (EXP 5, member of nuclear transport family). As with the other nuclear transport receptor, EXP 5 binds cooperatively to its cargo and the GTP-bound form of the cofactor Ran in the nucleus and releases the cargo following the hydrolysis of GTP in the cytoplasm. EXP 5 recognizes the >14 bp dsRNA stem along with a short 3′ overhang (1–8 nt) [7]. The 3rd step is cytoplasmic processing by the Dicer, pre-miRNA in the cytoplasm is typically further processed by the Dicer and transactivation-response RNA-binding protein (TRBP) RNase III enzyme complex to form the mature double-stranded ~22-nucleotide miRNA. Finally, the 4th step is argonaute loading, Argonaute proteins then unwind the miRNA duplex and facilitate incorporation of the miRNA-targeting strand (also known as the guide strand) into the AGO-containing RNA-induced silencing complex (RISC). The RISC-miRNA assembly is then guided to specific target sequences in mRNAs. The initial recognition of mRNAs by the RISC-miRNA complex is driven primarily by Watson-Crick base-pairing of nucleotides 2 to 8 in the mature miRNA (seed sequence) with specific mRNA target sequences chiefly located in the 3′ untranslated region, and additional base-pairing affords greater affinity and targeting efficiency [8].

## 3. Regulation of miRNAs Biogenesis

Precise control of miRNA levels is crucial to maintain normal cellular functions, and dysregulation of miRNA is often associated with human diseases, such as cancer [9].

### 3.1. Regulation at Transcriptional Level

Transcription is a major point of regulation in miRNA biogenesis. Numerous Pol II-associated transcription factors are involved in transcriptional control of miRNA genes. For instance, myogenic transcription factors, such as myogenin and myoblast determination 1 (MyoD1), bind upstream of miR-1 and miR-133 loci and induce the transcription of these miRNAs during myogenesis. Some miRNAs are under the control of tumour-suppressive or oncogenic transcription factors. The tumour suppressor p53 activates the miR-34 family of miRNAs [10], whereas the oncogenic protein MyC transactivates or represses a number of miRNAs that are involved in the cell cycle and apoptosis [11]. 

### 3.2. Regulation at Posttranscriptional Level

Drosha processing is also another important point of regulation. miR-21 is induced in response to bone morphogenetic protein (BMP)/transforming growth factor-*β* (TGF-*β*) signaling without transcriptional activation [12]. It was proposed that SMAD proteins activated by BMP/TGF-*β* interact with Drosha and DDX5 (also known as p68) to stimulate Drosha processing, although the detailed mechanism for this remains unclear. The let-7 miRNAs show interesting expression patterns. The primary transcript of let-7 (pri-let-7) is expressed in both undifferentiated and differentiated ES cells, whereas mature let-7 is detected only in differentiated cells, indicating that let-7 might be posttranscriptionally controlled [13]. Recent studies show that an RNA-binding protein, LIN28, is responsible for the suppression of let-7 biogenesis. Several different mechanisms of LIN28 action have been proposed: blockage of Drosha processing interference [14] with Dicer processing and terminal uridylation of pre-let-7 [14]. RNA editing is another possible way of regulating miRNA biogenesis. The alteration of adenines to inosines, a process that is mediated by adenine deaminases (ADARs), has been observed in miR-142 [15] and miR-151 [16]. Because the modified pri-miRNAs or pre-miRNAs become poor substrates of RNase III proteins, editing of the precursor can interfere with miRNA processing. Editing can also change the target specificity of the miRNA if it occurs in miRNA sequences [17]. 

### 3.3. Feedback Circuits in miRNA Networks

Two types of feedback circuits are frequently observed: single-negative feedback and double-negative feedback. The levels of Drosha and Dicer are controlled by single-negative feedback to maintain the homeostasis of miRNA production [18]. Drosha constitutes a regulatory circuit together with DGCR8; Drosha downregulates DGCR8 by cleaving *DGCR8* mRNA, whereas DGCR8 upregulates Drosha through protein stabilization. Double-negative feedback control is also often used as an effective genetic switch of specific miRNAs during differentiation. An interesting example is the conserved loop that involves let-7 and LIN28. miRNA let-7 suppresses LIN28 protein synthesis, whereas LIN28 blocks let-7 maturation. The miR-200 family and the transcriptional repressors Zeb1 and Zeb2 also constitute a double-negative feedback loop that functions in epithelial-mesenchymal transition program (EMT) [19].

## 4. EMT in Renal Fibrosis

**Figure 2:**
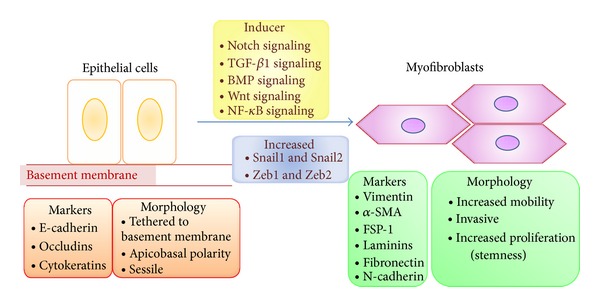
Biochemical changes during EMT in fibrosis. Repression of the transcription factors Snail1, Snail2, Zeb1, and Zeb2 is important for the maintenance of epithelial morphology. Several factors that are upregulated in the context of inflammation, including nuclear factor-*κ*B (NF-*κ*B), TGF-*β*1, bone morphogenetic proteins (BMPs), Wnt, and Notch signaling proteins, can activate the Snail-Zeb pathway, leading to mesenchymal differentiation in these cells. FSP-1: fibroblast-specific protein-1.

EMT involves a series of changes through which epithelial cells lose their epithelial characteristics and acquire properties typical of mesenchymal cells. EMT facilitates cell movement and the generation of new tissue types during development and also contributes to the pathogenesis of disease. Earlier the role of EMT in renal fibrosis was discussed in the review [20].  Figure 2 displayed unique phenotypes of epithelial and mesenchymal cells. Epithelial cells are normally associated tightly with their neighbors, which inhibit their potential for movement and dissociation from the epithelial layer. Epithelia contour the cavities and surfaces of organs throughout the body and also form many glands. In contrast, mesenchymal cells do not form a regular layer of cells or specialized intercellular adhesion complexes. Mesenchymal cells are elongated in shape relative to epithelial cells and exhibit end-to-end polarity and focal adhesions, allowing for increased migratory capacity. Although mesenchymal cells may be polarized when migrating or interacting with neighboring cells, they lack the typical apical-basal polarity seen in epithelia. Moreover, mesenchymal cells migrate easily within tissues individually or collectively by forming a chain of migrating cells. Mesenchymal cells are essential for development as they can migrate large distances across the embryo to give rise to a particular organ. In the adult, the main function of fibroblasts, prototypical mesenchymal cells that exist in many tissues, is to maintain structural integrity by secreting extracellular matrix (ECM). Dr. Kalluri proposed classification of EMT into following three subtypes based on context [21]. Type 1 EMT involves the transition of primordial epithelial cells into motile mesenchymal cells and is associated with the generation of diverse cell types during embryonic development and organogenesis. These type 1 EMTs neither cause fibrosis nor induce invasion, and, in many cases, the mesenchymal cells that are generated later undergo MET to give rise to secondary epithelia. Type 2 EMT involves transition of secondary epithelial cells to tissue fibroblasts and is associated with wound healing, tissue regeneration, and organ fibrosis. In contrast to type 1, type 2 EMT is induced in response to inflammation but stops once inflammation is attenuated, especially during wound healing and tissue regeneration [22]. During organ fibrosis, type 2 EMT continues to respond to persistent inflammation, resulting in tissue destruction [22]. Type 3 EMT occurs in carcinoma cells that have formed solid tumors and is associated with their transition to metastatic tumor cells that have the potential to migrate through the bloodstream and, in some cases, form secondary tumors at other sites through mesenchymal epithelial transition (MET) [23]. Fibroblast-specific protein 1 (FSP-1; also known as S100A4 and MTS-1), an S100 class of cytoskeletal protein, *α*-SMA, and collagen I have provided reliable markers to characterize the mesenchymal products generated by the EMTs that occur during the development of fibrosis in various organs [21]. These markers, along with discoidin domain receptor tyrosine kinase 2 (DDR2), vimentin, and desmin, have been used to identify epithelial cells of the kidney, liver, lung, and intestine that are in the midst of undergoing an EMT associated with chronic inflammation. Such cells continued to exhibit epithelial-specific morphology and molecular markers, such as cytokeratin and E-cadherin, but showed concomitant expression of the FSP-1 mesenchymal marker and *α*-SMA. Such cells are likely to represent the intermediate stages of EMT, when epithelial markers continue to be expressed, but new mesenchymal markers have already been acquired. The behavior of these cells provided one of the first indications that epithelial cells under inflammatory stresses can advance to various extents through an EMT, creating the notion of “partial EMTs.” Eventually, these cells leave the epithelial layer, negotiate their way through the underlying basement membrane, and accumulate in the interstitium of the tissue, where they ultimately shed all of their epithelial markers and gain a fully fibroblastic phenotype. Inflammatory injury to the mouse kidney can result in the recruitment of a diverse array of cells that can trigger an EMT through their release of growth factors, such as TGF-*β*, PDGF, EGF, and FGF-2 [21]. Most prominent among these cells are macrophages and activated resident fibroblasts that accumulate at the site of injury and release these growth factors. In addition, these cells release chemokines and MMPs, notably MMP-2, MMP-3, and MMP-9. The significance of TGF-*β*-induced EMT for progression of organ fibrosis has been demonstrated in studies using BMP-7, an antagonist of TGF-*β* signaling, in mouse models of kidney, liver, billiard tract, lung, and intestinal fibrosis [24]. BMP-7 functions as an endogenous inhibitor of TGF-*β*-induced EMT [24]. Among other effects, it reverses the TGF-*β*-induced loss of the key epithelial protein, E-cadherin. Restoration of E-cadherin levels by BMP-7 is mediated via its cognate receptors, activin like kinase-2/-3/-6 (ALK-2/-3/-6), and downstream transcription factors smads [24]. Systemic administration of recombinant BMP-7 to mice with severe fibrosis resulted in reversal of EMT and repair of damaged epithelial structures, with repopulation of healthy epithelial cells, all presumably mediated via an MET. This reversal was also associated with restoration of organ function, a substantial decrease in FSP-1+ and *α*-SMA+ interstitial fibroblasts, and the de novo activation of BMP-7 signaling [24]. However, these different EMT programs may be induced and regulated by a common set of stimuli, signal transduction pathways, transcription factors, and posttranslational regulations [22]. 

### 4.1. miRNAs in EMT

Genome-wide analysis for miRNAs has revealed that the miR200 family and miR205 are highly associated with EMT [25]. This change is reflected in a strong correlation between the expression of the miR200 family and E-cadherin across numerous cell lines and epithelial tissues [25, 26]. The miR200 family binds to the 3′ UTRs of RNA and suppresses the expression of Zeb1 and SIP1, which repress E-cadherin. The miR200 family is thereby capable of enforcing epithelial phenotypes. Additional EMT-related downstream targets of the miR200 family have been identified: miR141 inhibits TGF-*β*2 [26] and miR200a suppresses *β*-catenin (CTNNB1) [27]. miRNAs are also associated with the TGF-*β* signaling pathway. The expression of miR155 increases during TGF-*β*-induced EMT in mammary epithelial cells through smad4-mediated transcriptional upregulation and facilitates loss of cell polarity and tight junctions [28, 29]. Moreover, epithelial cells expressing miR155 responded more rapidly to TGF-*β*. A key downstream target of miR155 is RhoA, which plays a role in the formation and stabilization of cell junctions. RhoA contains three conserved regions that may serve as binding sites for miR155 [28]. These data suggest that miR155 may provide further inhibitory effects on RhoA during EMT, in addition to TGF-*β*-mediated ubiquitination and degradation. The expression levels of miR29a and miR21 also are increased upon TGF-*β*-induced EMT in mammary epithelial cells [28], although their role in EMT has not been completely elucidated. Overexpression of miR29a suppresses the expression of tristetraprolin (known as zinc finger protein 36 homolog, ZFP36) and leads to EMT in cooperation with the Ras signaling pathway [29].

### 4.2. Regulation of EMT

It was recently shown that miR9 directly targets the mRNA encoding E-cadherin [30]. Ectopic expression of miR9 led to EMT in human mammary epithelial cells [31]. Moreover, a significant number of breast carcinoma cells located at the edge of miR9-expressing tumors expressed mesenchymal markers including vimentin, whereas few cells located in intratumoral regions were vimentin-positive, suggesting that miR9 may sensitize cells to EMT-inducing signals from the tumor microenvironment [30]. The EMT-inducing transcription factors have recently emerged as transcriptional regulators of miRNAs. miR21 is highly expressed in various tumors and known to induce metastasis through EMT. The promoter regions of miR21 include consensus E-box sequences that serve as binding sites for Zeb1 [31]. Binding of Zeb1 induces transcription of miR21 and also blocks bone morphogenetic protein- (BMP-)6-mediated inhibition of EMT in breast cancer cells [31]. 

## 5. EndMT in Renal Fibrosis

**Figure 3:**
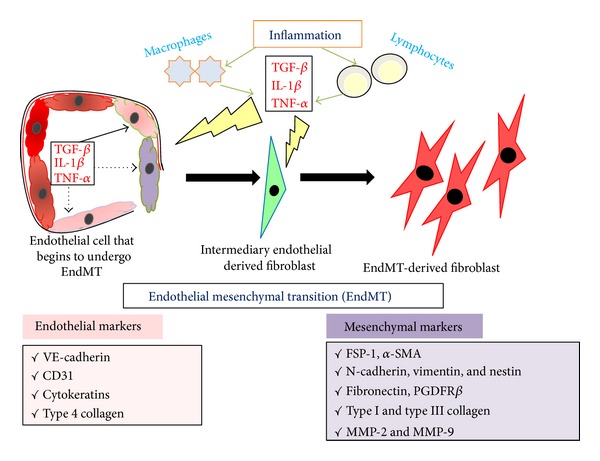
Biochemical changes during EndMT program. The EndMT program causes decreased expression of endothelial markers VE-cadherin, CD31, cytokeratins, and type 4 collagen and a gain of mesenchymal markers FSP-1, *α*SMA, N-cadherin, vimentin, fibronectin, type I and type III collagen, and MMP-2 and MMP-9. FSP-1: fibroblast-specific protein-1; *α*-SMA: *α*-smooth muscle actin; and MMP: matrix metalloproteinase.

Vascular endothelial cells share several common traits with epithelial cells and can generate fibroblasts by undergoing a phenotypic transition similar to EMT, referred to as endothelial-mesenchymal transition (EndMT). EndMT is characterized by the loss of endothelial markers including CD31 and vascular endothelial cadherin (VE-cadherin) and the expression of mesenchymal proteins including *α*-smooth muscle actin (*α*SMA) [32]. EndMT contributes to cardiac fibrogenesis which results in progressive stiffening of the ventricular walls, loss of contractility, and abnormalities in cardiac conductance [32]. EndMT is also involved in pulmonary fibrosis, idiopathic hypertension [33], and corneal fibrosis [34]. Many growth factors and signaling pathways that govern EMT also regulate EndMT in the embryonic heart and during cardiac fibrosis. However, as compared to EMT, relatively little is known about EndMT. Earlier role of EndMT in renal fibrosis was discussed and reviewed by many researchers [35, 36]. In the adult organism, pathological conditions such as injury, inflammation, or aging can awaken EndMT and induce the fibrosis of the involved organs. The EndMT program has also been suggested to contribute to the development and progression of cardiac fibrosis, pulmonary fibrosis, hepatic fibrosis, corneal fibrosis, intestinal fibrosis, and wound healing in addition to renal fibrosis [37]. Zeisberg et al., 2008, designed and conducted a landmark experiment that first confirmed the contribution of EndMT in renal fibrosis in three mouse models, unilateral ureteral obstruction (UUO; a model used to study progressive tubulointerstitial fibrosis), streptozotocin- (STZ-) induced diabetic nephropathy, and *α*3 chain of collagen type 4 (COL4A3) knockout mice (a mouse model for Alport syndrome). They found that a considerable proportion of myofibroblasts coexpress the endothelium marker CD31, also known as platelet endothelial cell adhesion molecule-1, and the (myo) fibroblast markers *α*SMA and fibroblast-specific protein-1 (FSP-1, also known as S100A4) in all three models [38]. Furthermore, they analyzed the kidneys 6 months after a single injection of STZ in CD1 mice, which exhibited progressive glomerular sclerosis and tubulointerstitial fibrosis. A double-immunolabeling experiment demonstrated that approximately 40% of all FSP-1 (+) and 50% of *α*SMA (+) stromal cells in STZ kidneys were also CD31-positive [38]. In the kidneys of 22-week-old COL4A3 knockout mice, 45% of all *α*SMA-positive fibroblasts and 60% of all FSP-1-positive fibroblasts were CD31-positive, suggesting that these fibroblasts are likely of endothelial origin and that EndMT may contribute substantially to the accumulation of fibroblasts in the development and progression of renal fibrosis [38]. Li et al., 2009, also confirmed that EndMT occurs and contributes to the generation of myofibroblasts in early diabetic renal fibrosis. Using endothelial-lineage tracing with Tie2-cre, LoxP-enhanced green fluorescent protein (EGFP) transgenic mice, they identified a significant number of interstitial *α*SMA-positive cells (myofibroblasts) of an endothelial origin in the fibrotic kidneys from mice with STZ-induced diabetic nephropathy [39]. ECs line the entire circulatory and lymphatic system, forming the inner lining of blood vessels and lymphatic vessels. These cells, which are anatomically similar to squamous epithelium, express apical-basal polarity and are tightly bound by adherens junctions and tight junctions [40]. These cells demonstrate a disparate set of biomarkers including VE-cadherin, CD31, TIE1, TIE2, von Willebrand factor (vWF), and cytokeratins. Similar to EMT, during EndMT, ECs lose their adhesion and apical-basal polarity to form highly invasive, migratory, spindle-shaped, elongated mesenchymal cells. Biochemical changes accompany these distinct changes in cell polarity and morphology, including the decreased expression of endothelial markers and the acquisition of mesenchymal markers (FSP-1, *α*SMA, SM22*α*, N-cadherin, fibronectin, vimentin, types I and III collagen, nestin, CD73, MMP-2, and MMP-9) [40] (Figure 3). 

### 5.1. miRNAs in EndMT

Indirectly, miRNAs also upregulate many genes via suppression of their repressor molecules. The expression of both primary and mature miRNA-21 (miR-21), especially the latter, was upregulated by TGF-*β* by silencing phosphatase and tensin homolog (PTEN) and activating the Akt pathway in EndMT *in vitro* (HUVECs) and *in vivo* (heart) [41]. smad3 signaling increases the expression of miR-21 in the kidney to promote renal fibrosis in response to TGF-*β* whereas smad2 negatively regulates the posttranscriptional modification of miR-21 [42]. miR-23 inhibits TGF-*β*-induced EndMT in mouse ECs, and miR-23 in the embryonic heart is required to restrict endocardial cushion formation by inhibiting hyaluronic acid synthase 2 (Has2) expression and extracellular hyaluronic acid production in Zebrafish dicer mutants [43]. Using the miRNA array analysis, Ghosh et al. [44] found that although miR-125b, let-7c, let-7g, miR-21, miR-30b, and miR-195 were significantly elevated during EndMT, the levels of miR-122a, miR-127, miR-196, and miR-375 were significantly downregulated. MiR-125b is approximately 4-fold higher in EndMT-derived fibroblast compared with MCECs [44]. The level of cellular p53, the major target of miR-125b and a known negative modulator of TGF-*β*-induced profibrotic signaling, was significantly suppressed with an elevated level of *α*SMA [44]. Blockade of FGF signaling induced EndMT program can be mimicked by the let-7b or let-7c miRNA inhibition. Although these studies were mostly performed in the heart, MCECs, or HUVECs, they still suggest that the specific suppression of upregulated miRNAs, such as miR-21, or the specific overexpression of downregulated miRNAs, such as miR-125b, may be a viable approach to blocking the induction of EndMT in a wide variety of organs. 

## 6. miRNAs in Kidney Disease and Diabetic Nephropathy

**Figure 4:**
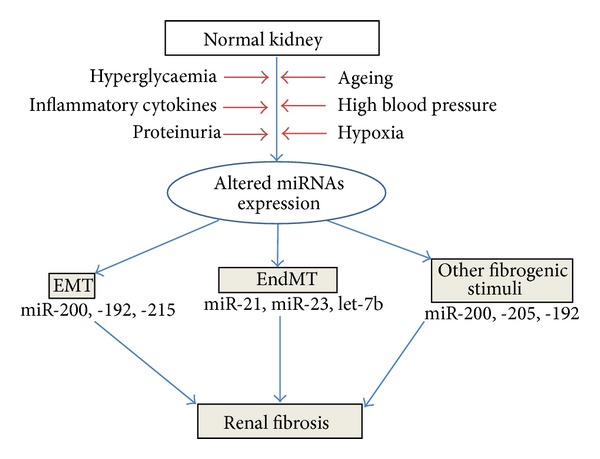
Implications of miRNAs in renal fibrosis.

Diabetic nephropathy is a progressive kidney disease and a major debilitating complication of both type 1 and type 2 diabetes that can lead to end-stage renal disease (ESRD) and related cardiovascular disorders. Absence or lower levels of particular miRNAs in the kidney compared with other organs may permit renal specific expression of target proteins that are important for kidney functions [45].  Figure 4 depicts the connection between the role of miRNAs and kidney fibrosis. Altered expression of miRNAs causes renal fibrosis by inducing EMT, EndMT, and other fibrogenic stimuli. The accumulative effects of hyperglycaemia, inflammatory cytokines, proteinuria, ageing, high blood pressure, and hypoxia result into alteration of miRNAs expression profiles. The altered miRNAs level causes the initiation of such transition program in normal kidney, finally fibrosis. Some of the miRNAs that are more abundant in the kidney compared with other organs include miR-192, miR-194, miR-204, miR-215, and miR-216. A critical role of miRNA regulation in the progression of glomerular and tubular damage and the development of proteinuria been suggested by studies in mice with podocyte-specific deletion of Dicer [46]. There was a rapid progression of renal disease with initial development of albuminuria followed by pathological features of glomerulosclerosis and tubulointerstitial fibrosis. It is likely that these phenotypes are due to the global loss of miRNAs because of Dicer deletion, but, given multiple miRNAs and their myriad targets, the precise pathways responsible require identification. These investigators also identified specific miRNA changes, for example, the downregulation of the miR-30 family when Dicer was deleted. Of relevance, the miR-30 family was found to target connective tissue growth factor, a profibrotic molecule that is also downstream of transforming growth factor (TGF)-*β* [47]. Thus, the targets of these miRNAs may regulate critical glomerular and podocyte functions. These findings have also been complemented by an elegant study revealing a developmental role for the miR-30 family during pronephric kidney development in *Xenopus* [48]. Sun et al. [49] identified five miRNAs (-192, -194, -204, -215, and -216) that were highly expressed in human and mouse kidney using miRNA microarray. A recent report using new proteomic approaches to profile and identify miRNA targets demonstrated that miRNAs repress their targets at both the mRNA and translational levels and that the effects are mostly relatively mild [50]. The role of miR-192 remains controversial and highlights the complex nature of miRNA research. Kato et al. [51] observed increased renal expression of miR-192 in streptozotocin- (STZ-) induced diabetes and in the db/db mouse and demonstrated that transforming growth factor (TGF-*β*1) upregulated miR-192 in mesangial cells (MCs). miR-192 repressed the translation of Zeb2, a transcriptional repressor that binds to the E-box in the collagen 1*α*2 (col1*α*2) gene. They proposed that miR-192 repressed Zeb2 and resulted in increased col1*α*2 expression *in vitro* and contributed to increased collagen deposition *in vivo*. These data suggest a role for miR-192 in the development of the matrix accumulation observed in DN. It is interesting that the expression of miR-192 was increased by TGF-*β* in mouse MCs (mesangial cells), whereas, conversely, the expression of its target, Zeb2, was decreased [51]. This also paralleled the increased Col1 *α*2 and TGF-*β* expression [51]. These results suggested that the increase in TGF-*βin vivo* in diabetic glomeruli and *in vitro* in MCs can induce miR-192 expression, which can target and downregulate Zeb2 thereby to increase Col1 *α*2. This is supported by the report showing that miR-192 is upregulated in human MCs treated with high glucose [51]. TGF-*β* induced downregulation of Zeb2 (*via* miR-192) and Zeb1 (*via* potentially another miRNA) can cooperate to enhance Col1 *α*2 expression *via* de-repression at E-box elements [51]. In contrast to the above, other reports suggest the relationship between miR-192 and renal fibrosis may be more complicated. Krupa et al. [52] identified two miRNAs in human renal biopsies, the expression of which differed by more than twofold between progressors and nonprogressors with respect to DN, the greatest change occurring in miR-192 which was significantly lower in patients with advanced DN, correlating with tubulointerstitial fibrosis and low glomerular filtration rate. They also reported, in contrast to the Kato et al. [51] study in MCs, that TGF-*β*1 decreased expression of miR-192 in cultured proximal tubular cells (PTCs). These investigators concluded that a decrease in miR-192 is associated with increased renal fibrosis *in vivo*. Interestingly, connective tissue growth factor (CTGF) treatment also resulted in fibrogenesis but caused the induction of miR-192/215 and, consequently, decreased Zeb2 and increased E-cadherin. The contrasting findings above highlight the complex nature of miRNA research. Some of the differences may relate to models and/or experimental conditions; however, one often overlooked explanation is that some effects of miRNAs and inhibitors are likely to be indirect in nature. A recent report also showed that BMP6-induced miR-192 decreases the expression of Zeb1 in breast cancer cells [53]. Thus, TGF-*β* induced increase in the expression of key miRNAs (miR-192 and miR-200 family members) might coordinately downregulate E-box repressors Zeb1 and Zeb2 to increase Col1*α*2 expression in MCs related to the pathogenesis of DN. The proximal promoter of the *Col1a2* gene responds to TGF-*βvia* smads and SP1. Conversely, the downregulation of Zeb1 and Zeb2 by TGF-*βvia* miR-200 family and miR-192 can affect upstream E-box regions. Because E-boxes are present in the upstream genomic regions of the miR-200 family, miR-200 family members may themselves be regulated by Zeb1 and Zeb2 [54]. It is possible that the miR-200 family upregulated by TGF-*β* or in diabetic glomeruli under early stages of the disease can also regulate collagen expression related to diabetic kidney disease by targeting and downregulating E-box repressors. miR-192 might initiate signaling from TGF-*β* to upregulate miR-200 family members, which subsequently could amplify the signaling by further regulating themselves through down regulation of E-box repressors. Such events could lead to progressive renal dysfunction under pathologic conditions such as diabetes, in which TGF-*β* levels are enhanced. Conversely, there are several reports that miR-200 family members and miR-192 can be suppressed by TGF-*β*, and this promotes epithelial-to-mesenchymal transition (EMT) in cancer and other kidney-derived epithelial cell lines *via* subsequent upregulation of targets Zeb1 and Zeb2 to repress E-cadherin [54, 55].

## 7. Prospective

The discovery of miRNAs in 1993 in the nematode made the tremendous revolution in the field of RNA world. There are several major challenges in exploring the role of miRNAs in kidney diseases. Now miRNA-based therapeutics has already entered Phase 2 clinical trials. miR-122 antagonists are the indicator of hepatitis C virus and now in the Phase 2 clinical trials [56]. miR-208/499 antagonists are the indicator for chronic failure and now in the preclinical development. Likewise miR-195 antagonists are also in preclinical development, used for the indicator of postmyocardial infarction remodeling. Some of the miRNAs (miR-34 and miR-7) are in preclinical development for the miRNA replacement therapy of cancer [57, 58]. This rapid progress from discovery to development reflects the importance of miRNAs as critical regulators in human disease and holds the promise of yielding a new class of therapeutics that could represent an attractive addition to the current drug pipeline of Big Pharma. Most importantly many fundamental questions remain regarding miRNA biology. The mechanism of regulation of miRNA is not completely clear. While many miRNAs are located within the intron of the host gene, their expression does not correlate perfectly with that of host genes suggesting further, posttranscriptional regulation [59]. Furthermore, the use of the miRNAs as therapeutic agents is attractive but faces considerable challenges, including development of safe and reliable organ and cell-specific delivery system, avoidance of toxicity derived from off-target effects and from activation of the innate and adaptive immune response. Current health statistics suggest that nearly 45% of all deaths in the western world can be attributed to some types of chronic fibro-proliferative disease [60]. EMT and EndMT have become a key topic in the study of organ fibrosis, since stressed and injured epithelium can give rise to myofibroblasts and thereby contribute to fibrogenesis therapeutics for fibrosis. The participation of EMT and EndMT in the pathogenesis of various fibrotic disorders requires confirmation and validation from further studies of human clinical pathological conditions. Future efforts should also be devoted to further understanding of the molecular mechanisms and the regulatory controls involved in these processes including miRNA regulation. These efforts would eventually lead to the development of novel therapeutic approaches for these incurable and often devastating disorders by targeting miRNAs.

## Abbreviations

ADARs: Adenine deaminases
*α*SMA: *α*-Smooth muscle actin
COL4A3: *α*3 chain of collagen type 4
BMP: Bone morphogenetic protein
CTGF: Connective tissue growth factor
DN: Diabetic nephropathy
DGCR8: DiGeorge syndrome critical region gene 8 protein
EndMT: Endothelial mesenchymal transition
EMT: Epithelial mesenchymal transition
ECM: Extracellular matrix
EXP 5: Exportin 5 (member of nuclear transport family)
EGFP: Enhanced green fluorescent protein (EGFP)
ESRD: End-stage renal disease
FSP-1: Fibroblast-specific protein-1
FGF: Fibroblast growth factor
microRNAs: miRNAs
MyoD1: myoblast determination 1
MET: Mesenchymal epithelial transition
MCs: Mesangial cells
MMP: Matrix metalloproteinase
PTEN: Phosphatase and tensin homolog
PTCs: Proximal tubular cells
RISC: RNA-induced silencing complex
TRBP: Transactivation-response RNA-binding protein
TGF-*β*: Transforming growth factor-*β*
UTRs: Untranslated regions
VE-cadherin: Vascular endothelial cadherin.
